# Nuclear anomalies in the buccal cells of calcite factory workers

**DOI:** 10.1590/S1415-47572010005000021

**Published:** 2010-06-01

**Authors:** Songül Budak Diler, Serap Ergene

**Affiliations:** 1 University of Nigde, Science and Letters, NigdeTurkey; 2 University of Mersin, Science and Letters, MersinTurkey

**Keywords:** calcite, exfoliated buccal cells, micronucleus (MN), genotoxicity

## Abstract

The micronucleus (MN) assay on exfoliated buccal cells is a useful and minimally invasive method for monitoring genetic damage in humans. To determine the genotoxic effects of calcite dust that forms during processing, MN assay was carried out in exfoliated buccal cells of 50 (25 smokers and 25 non-smokers) calcite factory workers and 50 (25 smokers and 25 non-smokers) age- and sex-matched control subjects. Frequencies of nuclear abnormalities (NA) other than micronuclei, such as binucleates, karyorrhexis, karyolysis and ‘broken eggs', were also evaluated. Micronuclei and the other aforementioned anomalies were analysed by two way analysis of covariance. The linear correlations between the types of micronucleus and nuclear abnormalities were determined by Spearman's Rho. There was a positive correlation between micronuclei and other types of nuclear abnormalities in accordance with the Spearman's Rho test. Results showed statistically significant difference between calcite fabric workers and control groups. MN and NA frequencies in calcite fabric workers were significantly higher than those in control groups (p < 0.05). The results of this study indicate that calcite fabric workers are under risk of significant cytogenetic damage.

The chemical formula of calcite that forms limestone is CaCO_3_ (Cotton and Wilkinson, 1988). Generally it does not exist in pure form in nature. It forms a complex with other chemicals (SiO_2_, Al_2_O_3_, FeO_3_, CaO, MgO, SO_3_, Na_2_O, K_2_O, CO_2_, Fe^+^_2_ (Goldsmith *et al.*, 1962; Jeong and Youngsin, 2006).

In Turkey, limestone (deposits) reserves have a high percentage of CaCO_3_ (96.8% max.) together with other substances, such as MgCO_3_ (1% max), Fe_2_O_3_ (0.3% max), acid non-soluble material (0.02% max) and water (0.4% max). The amount of silica and impurities from iron compounds are very low (Böke *et al.*, 1999; Dilsiz, 2002).

After being ground into a fine powder, calcite is widely used in industry for making paint, paper and plastic adhesives, as well as a filling substance in food, chemical and construction products (Yekeler and Ozkan, 2002).

The harmful effects of mineral dust in various forms on human health have already been demonstrated (Guthrie, 1992; Dong *et al.*, 2006; Prince *et al.*, 2008), notably the dust from iron ore was found to be toxic by genotoxicity tests (Dönbak *et al.*, 2005). In addition, the dust of the calcite mineral was found to cause lung damage (Jaunarajs and Liebling, 1972; Zhang and Huang, 2005; Erren *et al.*, 2007). On investigating the genotoxic effects of environmental factors on human nuclei, the micronucleus (MN) test has been employed on buccal epithelial cells (Sarto *et al.*, 1987; Tolbert *et al.*, 1992; Levine *et al.*, 1997), The MN test, which is scientifically approved, is important in demonstrating the genotoxic effects of harmful substances on human health (Nersesyan, 2005; Nersesyan *et al.*, 2006; Fenech *et al.*, 2007), such as in measuring genotoxicity in petrol station employees (Çelik *et al.*, 2003; Benites *et al.*, 2006), agricultural workers (Pastor *et al.*, 2001), cigarette smokers and tobacco users (Özkul *et al.*, 1997; Besaratinia *et al.*, 2000; Proia *et al.*, 2006**)**. In addition, it has been used in workers exposed to pesticides (Pastor *et al.*, 2002), polycyclic aromatic hydrocarbons (Karahalil *et al.*, 1999), timber dust (Çelik and Kanik, 2006), ozone and cancer patients (Bloching *et al.*, 2000; Chen *et al.*, 2006).

The aim of our study was to determine the effects of calcite dust formed during its processing on the health of employees of calcite factories by employing the MN test on buccal epithelial cells, which has not been performed so far in any other study in this context.

The study was carried out with 50 calcite factory-workers, (25 smokers and 25 non-smokers) working in the town of Nigde. The control group consisted of 50 healthy men (25 smokers and 25 non-smokers) working on the Nigde University campus, with no known previous occupational exposure to genotoxic substances. The questionnaire covered standard demographic (age, gender, etc.), lifestyle (smoking, alcohol consumption, etc.) and occupational (working hours/day, period of exposure, etc.) aspects. There were no significant differences as regards lifestyle and personal factors in participants of the study groups.

Buccal cell samples were collected at the end of work-shifts. Subjects were required to rinse their mouths with water before sampling. Exfoliated epithelial cells of buccal mucosa were obtained by scraping the middle part of the inner cheek with a wooden spatula. The epithelial cells collected from buccal mucosa were smeared onto clean microscope glass slides. The slides were air-dried and fixed with cold 100% methanol. The slides were incubated at 37 °C overnight and then stained with Giemsa (Stich and Rosin, 1984; Acar *et al.*, 2001).

A light microscope using 100 X magnification on coded slides was used for MN analysis. Four replicate slides were prepared for each subject and 1000 cells evaluated per slide to determine MN frequency. Thus, at least 4000 cells per person were scored for every four slides.

MN and other nuclear abnormalities were classified according to Tolbert *et al.* (1992). MNs must satisfy the following conditions: a) consist of nuclear material; b) be completely separated from the parent nucleus; c) be less than 1/3 of the diameter of associated nuclei; d) be smooth, oval- or round-shaped; e) be on the same plane of focus and f) be of the same color, texture and refraction as the main nucleus. Cells with two nuclei were considered to be binucleate. Besides MN, other nuclear anomalies, such as karyorrhexis (nuclear disintegration), karyolysis (dissolution of nucleus) and ‘broken eggs' (nuclei that appeared cinched) were recorded separately according to Tolbert *et al.* (1991).

The statistical analyses were performed using the SPSS software, version 11.5 (SPSS, Chicago,IL). All the data were expressed as the mean ± standard error of the mean. MNs and other nuclear abnormalities (binucleates, karyorrhexis, karyolysis and ‘broken eggs') were analysed by two way analysis of covariance in workers and smokers in order to delineate possible differences between these two groups. In all analyses, the age variable was include in the calculations as a covariant. Linear correlations of the various types of MN and nuclear abnormalities were determined by Spearman's Rho. The SPSS v11.5 programme package was used for statistical analysis. Results with p < 0,05 were considered significant.

A summary of dermographic characteristics of the groups studied appear in Table 1. 25 of the 50 members (50%) of both the study and control groups were smokers. As described in materials and methods, buccal epithelial samples were obtained from all the 100 involved subjects. 4000 cells from each were evaluated for micronuclei and other nuclear abnormalities (binucleates (BN), karyorrhexis (KR), karyolysis (KL), ‘broken eggs' (BE)), counted and statistically analyzed using SPSS. MN and the other anomalies were appraised through two way analysis of covariance in workers and smokers in order to delineate possible differences between the two groups. Differences in age were not statistically significant (p > 0.05). In terms of duration of work the difference between smokers and non-smokers was also not significant (p > 0.05) (Table 1). With respect to BE, the interaction for working and smoking was significant (p < 0.05), whereas the opposite was true for MN, BN, KR and KL (p > 0.05) (Table 2). For MN, BN and BE, there was a significant difference (p < 0.05) between smokers and non-smokers, whereas the opposite was the case for KR and KL (p > 0.05). There was also a significant difference between exposed workers and control groups as regards MN, BN, BE, KR and KL (p < 0.05) (Table 3). Furthermore, according to Spearman's Rho testing, the correlations between MN and other types of nuclear abnormalities were positive (Table 4).

The MN test is a very useful and efficient method for specifying buccal epithelial cell chromosomal abnormalities (Levine *et al.*, 1997; Holland *et al.*, 2008). Although studies on the genotoxicity of calcite mineral dust are very limited, the effect of this exposure on the lungs has been investigated more extensively and it was found to be damaging (Seldén *et al.*, 2001; Dogan, 2003; Özkurt *et al.*, 2003). At the same time it has been shown that some mineral dusts are factors in lung cancer risk (Wendlandt *et al.*, 2007; Dogan *et al.*, 2008).

The factor ‘smoking' was also taken into account, whereby it was found that nuclear abnormalities were higher in smokers, and previous studies in this regard confirm our findings (Özkul *et al.*, 1997; Proia *et al.*, 2006**)**. Other investigators used the buccal MN test and demonstrated that there was an increase in the incidence of nuclear abnormalities in alcohol consumers and workers exposed to environmental pollutants (Ramirez and Saldanha, 2002; Reis *et al.*, 2006**)**. Furthermore, on using the same assay in measuring micronuclei and nuclear abnormalities in timber workers exposed to wood dust, the difference between these and control subjects was found to be statistically significant (Çelik and Kanik, 2006). Similar genotoxic studies in coal mine workers demonstrated that the incidence of nuclear anomalies, including sister chromatid exchanges (SCEs) and chromosomal aberrations (CAs), in those exposed to coal dust, was higher than in controls (Dönbak *et al.*, 2005).

Our study too shows that there is an increase in MN and nuclear abnormalities in workers exposed to calcite dust when compared to on-exposed controls.

## Figures and Tables

**Table 1:** General characteristics of the groups studied.

Study group		n	Age (years)	Average no of cigarettes/day	Duration of employment (years)
Control	Smokers	25	29.00 ± 1.76	23	
	Non-smokers	25	32.36 ± 1.85		
	Total	50	30.68 ± 1.28	23	
Workers	Smokers	25	29.84 ± 1.06	18	3.24 ± 0.44
	Non-smokers	25	31.68 ± 0.99		4.00 ± 0.69
	Total	50	30.76 ± 0.73	18	3.62 ± 0.40

**Table 2:** The frequencies (‰) of micronuclei and other nuclear abnormalities in exfoliated buccal epithelial cells of control and exposed subjects.

Group		n	Micronuclei mean (a) ± SE	Nuclear abnormality			
				Binucleates mean (a) ± SE	Broken eggs mean (a) ± SE	Karyorrhexis mean (a) ± SE	Karyolysis mean (a) ± SE
Control	Smokers	25	11.41 ± 1.12	4.75 ± 0.88	3.52 ± 1.51	1.10 ± 0.25	1.23 ± 0.43
	Non-smokers	25	4.68 ± 1.11	3.42 ± 0.88	5.21 ± 1.51	1.05 ± 0.25	1.31 ± 0.43
Workers	Smokers	25	25.52 ± 1.11	11.28 ± 0.87	15.16 ± 1.50	3.05 ± 0.25	1.90 ± 0.43
	Non-smokers	25	15.19 ± 1.11	6.98 ± 0.87	4.09 ± 1.50	2.27 ± 0.25	3.10 ± 0.43

(a): Covariates appearing in the model are evaluated at the following values: Age = 30,9400.

**Table 3:** The mean effect (‰) of micronuclei and other nuclear abnormalities in exfoliated buccal epithelial cells of subjects from control groups, exposed workers, smokers and non-smokers.

Group	n	Micronuclei mean (a) ± SE	Nuclear abnormality			
			Binucleates mean (a) ± SE	Broken eggs mean (a) ± SE	Karyorrhexis mean (a) ± SE	Karyolysis mean (a) ± SE
Control	50	8.04 ± 0.78	4.08 ± 0.61	4.37 ± 1.06	1.07 ± 0.17	1.27 ± 0.30
Workers	50	20.36 ± 0.78	9.13 ± 0.61	9.62 ± 1.06	2.66 ± 0.17	3.50 ± 0.30
Smokers	50	18.47 ± 0.78	8.01 ± 0.61	10.19 ± 1.06	2.07 ± 0.17	2.57 ± 0.30
Non-smokers	50	9.93 ± 0.78	5.20 ± 0.61	3.81 ± 1.06	1.66 ± 0.17	2.20 ± 0.30

(a): Covariates appearing in the model are evaluated at the following values: Age = 30,9400.

**Table 4:** The correlation coefficient between MNs and different types of cytogenetic endpoints.

		MN	BN	BE	KL	KR
MN	Correlation Coefficient		0.643(**)	0.417(**)	0.528(**)	0.588(**)
	Sig. (2-tailed)		0.000	0.000	0.000	0.000
	N		100	100	100	100
BN	Correlation Coefficient	0.643(**)		0.624(**)	0.516(**)	0.615(**)
	Sig. (2-tailed)	0.000		0.000	0.000	0.000
	N	100		100	100	100
BE	Correlation Coefficient	0.417(**)	0.624(**)		0.218(*)	0.402(**)
	Sig. (2-tailed)	0.000	0.000		0.030	0.000
	N	100	100		100	100
KL	Correlation Coefficient	0.528(**)	0.516(**)	0.218(*)		0.688(**)
	Sig. (2-tailed)	0.000	0.000	0.030		0.000
	N	100	100	100		100
KR	Correlation Coefficient	0.588(**)	0.615(**)	0.402(**)	0.688(**)	
	Sig. (2-tailed)	0.000	0.000	0.000	0.000	
	N	100	100	100	100	

** Correlation is significant at the 0.01 level (2-tailed). * Correlation is significant at the 0.05 level (2-tailed).
